# Trends, Risk Factors, and Regional Disparities of Alzheimer's Disease and Dementia in Pakistan from 1990 to 2021: A Systematic Analysis of Global Burden of Disease

**DOI:** 10.1002/alz70860_103506

**Published:** 2025-12-23

**Authors:** Muhammad Safiullah, Muhammad Atif Bashir, Muhammad Ali, Muhammad Imaz Bhatti, Muhammad Nabeel Saddique, Anurag Jha, Izza Fatima

**Affiliations:** ^1^ King Edward Medical University, Lahore, Punjab, Pakistan; ^2^ Rawalpindi Medical University, Rawalpindi, Punjab, Pakistan

## Abstract

**Background:**

This study examined the burden of Alzheimer's disease and other dementias (ADOD) and attributable factors at the national and provincial levels in Pakistan.

**Method:**

We used the Global Burden of Diseases Study 2021 to estimate incidence, mortality rate, and disability‐adjusted life years (DALYs) for ADOD in Pakistan. Estimated annual percentage changes (APCs) and average annual percentage changes (AAPCs) were used to quantify the temporal trends from 1990 to 2021.

**Result:**

In 2021, Pakistan experienced the highest ADOD burden in South Asia after Bhutan. The age‐standardized incidence rates (ASIR) for ADOD had an overall decreasing trend from 1990‐2021. In 1990, the ASIR was 84.44 (95% UI: 73.08 to 97.02) and 78.80 (95% UI: 67.99 to 90.38) in 2021 (AAPC: ‐0.2235; 95% CI: ‐0.2250 to ‐0.2223). From 1990 to 2021, the age‐standardized death rates (ASDR) and age‐standardized DALYs rate showed increasing trends with an AAPC of 0.4355 (95% CI: 0.4204 to 0.4532) and 0.2232 (95% CI:0.2104 to 0.2380) respectively. The number of people with ADOD increased by 81.97% and DALYs associated with ADOD increased by 105.49%; most of these increases were explained by population aging. In subregion‐wise analysis, Islamabad had the highest age‐standardized rates, while Khyber Pakhtunkhwa had the lowest. Females aged 70 and above experienced the highest increase in the burden of ADOD. High fasting plasma glucose was the leading cause of ADOD‐related mortality in 2021.

**Conclusion:**

ADOD, a major concern, needs focused healthcare attention, especially for older female individuals. Metabolic risks should be prioritized for prevention, and to decrease ADOD related mortality.

